# Complex Ancient Genetic Structure and Cultural Transitions in Southern African Populations

**DOI:** 10.1534/genetics.116.189209

**Published:** 2016-11-10

**Authors:** Francesco Montinaro, George B. J. Busby, Miguel Gonzalez-Santos, Ockie Oosthuitzen, Erika Oosthuitzen, Paolo Anagnostou, Giovanni Destro-Bisol, Vincenzo L. Pascali, Cristian Capelli

**Affiliations:** * Department of Zoology, University of Oxford, OX1 3PS, UK; † Wellcome Trust Centre for Human Genetics, Oxford OX3 7BN, UK; ‡ School of Medicine, University of Namibia, Private Bag 13301 Windhoek, Namibia; § Dipartimento di Biologia Ambientale, Università “La Sapienza,” 00185 Rome, Italy; ** Istituto Italiano di Antropologia, 00185 Rome, Italy; †† Institute of Public Health, Catholic University, 00168 Rome, Italy

**Keywords:** Khoesan, sub-Saharan Africa, ancient structure, African prehistory

## Abstract

The characterization of the structure of southern African populations has been the subject of numerous genetic, medical, linguistic, archaeological, and anthropological investigations. Current diversity in the subcontinent is the result of complex events of genetic admixture and cultural contact between early inhabitants and migrants that arrived in the region over the last 2000 years. Here, we analyze 1856 individuals from 91 populations, comprising novel and published genotype data, to characterize the genetic ancestry profiles of 631 individuals from 51 southern African populations. Combining both local ancestry and allele frequency based analyses, we identify a tripartite, ancient, Khoesan-related genetic structure. This structure correlates neither with linguistic affiliation nor subsistence strategy, but with geography, revealing the importance of isolation-by-distance dynamics in the area. Fine-mapping of these components in southern African populations reveals admixture and cultural reversion involving several Khoesan groups, and highlights that Bantu speakers and Coloured individuals have different mixtures of these ancient ancestries.

SOUTHERN Africa is characterized by substantial spatial and diachronic cultural variation. Archaeologically, the prehistory of this part of the continent has been characterized by extended regional variation in lithic industries at the interface between the Middle and Later Stone Ages (Mitchell 2002). The recent arrival of pastoralism and agriculture further complicated the cultural profile of this region. Human and livestock remains document the appearance of herders in the region <2 KYA, and several disciplines have attempted to map the local dispersal of agro-pastoralist Bantu speaking populations during the last few centuries. The arrival of European colonists and the subsequent relocation of groups from Asia have added additional complexity to the history of the region. Extended variation can be also observed from a linguistic point of view. Bantu languages are the most commonly spoken in southern Africa, where they have been subdivided into Western and Southern in relation to their geographical distribution. Some of the non-Bantu languages spoken in southern Africa are characterized by click-sounds and are often referred to as Khoesan [here intended as a nongenealogical group of click-containing languages spoken by a variety of southern African herders and hunter-gatherers (Guldemann and Fehn 2014)]. These languages are classified into three major families (Blench 2006; Guldemann and Fehn 2014): the Kx’a, the Taa, and the Khoe-Kwadi, and are characterized by broad and overlapping geographic distributions. This cultural complexity extends also to the different subsistence economies implemented by groups who reside in this region, which include hunter-gathering, animal husbandry, and agriculture, plus various combinations of these strategies (Murdock 1981; Barnard 1992). From a genetic point of view, Africa hosts most of the worldwide genomic variability (Campbell and Tishkoff 2010), and some of the earliest branching Y chromosome and mitochondrial DNA lineages are located in the Southern part of the continent (Tishkoff *et al.* 2007; Batini *et al.* 2011; Rosa and Brehem 2011; Barbieri *et al.* 2013b). Due to their potential significance for the origin of modern humans, groups residing in southern Africa have attracted the attention of both geneticists and the general public (Batini *et al.* 2011; Pickrell *et al.* 2012; Schlebusch *et al.* 2012; Barbieri *et al.* 2013b; Gurdasani *et al.* 2015). Such interest has capitalized on the advent of new tools for genome analysis, which have contributed to a better characterization and understanding of the history of southern African populations (Henn *et al.* 2012; Pickrell *et al.* 2012, 2014; Schlebusch *et al.* 2012; Kim *et al.* 2014). Model-based analyses have demonstrated that populations located north of the Kalahari desert, such as Ju|’Hoan and !Xun, are characterized by a so-called *Northern* component, which is substantially different from that characterizing populations located to the south of the Kalahari (referred to as the *Southern* component (Pickrell *et al.* 2012; Schlebusch *et al.* 2012). However, in-depth analyses of Khoesan genetics have suggested a greater degree of complexity within Khoesan-speaking populations. For example, Schlebusch *et al.* (2012) highlighted the genetic peculiarity of G|ui and G||ana individuals when compared with Northern and Southern Khoesan (here referring to the geographic location of Khoesan speaking groups), while Petersen and collaborators (Petersen *et al.* 2013) suggested additional structure among Northern Khoesan populations (Ju|’Hoan and !Xun). In addition to this early structure, a signal of west Eurasian ancestry, which predates the arrival of Bantu-speaking farmers, has also been detected (Schlebusch *et al.* 2012; Pickrell *et al.* 2014).

Despite several investigations conducted in the past few years, we are still far from a detailed dissection of the genomic structure related to Khoesan speaking populations. Its exhaustive characterization is challenging due to the fact that various ancestral groups have overlapped over the last millennia, and that gene-flow has probably been common among groups. In this context, the legacy left by Khoesan in highly admixed groups such as southern African Bantu speakers and Coloured populations is far from clear, which makes the design and interpretation of regional genome-wide association studies challenging (Price *et al.* 2010; Rosenberg *et al.* 2010). Reconstruction of the ancestry profiles of these populations is further complicated by the fact that groups speaking different languages, and implementing different lifestyles, have been in contact for extended periods of time, prompting genetic and cultural exchange.

Here, to further dissect and clarify the genomic stratification of southern African populations, we analyze 1856 individuals from 91 populations using a combination of novel (59 individuals from seven populations) and published genome-wide SNP data. By applying a local ancestry deconvolution approach, we highlight previously unobserved complexity in the Khoesan-related genetic variation, and generate novel insight into the genetic history of the region. We provide evidence for the presence of at least three distinct Khoesan ancestral components, and reveal a substantial degree of admixture between Khoesan groups. Our fine dissection of the Khoesan-related legacy in highly admixed populations also reveals slight differences between Coloured and Bantu-speaking populations, possibly suggesting admixture with different Khoesan sources. Finally, we demonstrate that Khoesan-related structure is highly correlated with the geographic location of populations, but not with linguistic affiliations or subsistence strategies.

## Materials and Methods

### New data

We generated novel genotype data for 59 individuals from seven southern African populations collected in Namibia and Lesotho. Forty-four of these individuals from four Bantu speaking groups (MbukushuM, OwamboM, Kwangali, and Sotho), and a Khoesan-speaking group (NamaM), have been published previously (González-Santos *et al.* 2015), using a subset of the markers (∼2000). Eight individuals each from the Damara and Hai||om, collected in the Khorixas and Etosha areas of Namibia, respectively, are presented here for the first time. Detailed information about the collecting process and samples are available elsewhere (Marks *et al.* 2012, 2015; González-Santos *et al.* 2015). Full ethical approval for the collections was provided by the Oxford Tropical Research Ethics Committee (OxTREC), the Lesotho Ministry of Health and Social Welfare, the Lesotho Ministry of Local Government, the Lesotho Ministry of Tourism, Environment and Culture, and the Namibian Ministry of Health and Social Services. The Nama, Owambo, and Sotho populations were genotyped on the Illumina Human 610-Quad BeadChip (Illumina, San Diego, CA), while the Hai||om, Kwangali, Damara, and Mbukushu were genotyped on the Human Omni5-Quad BeadChip (Illumina, San Diego, CA).

### Existing datasets

**Figure 1:**
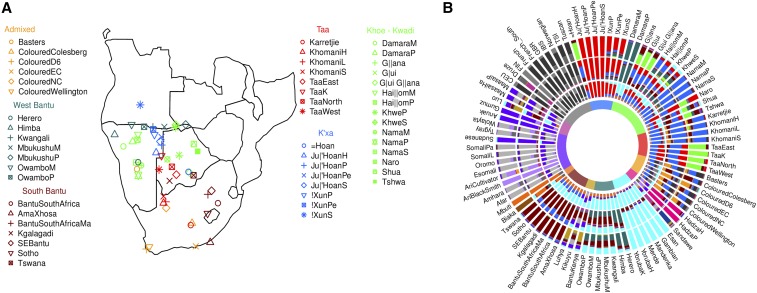
The genetic structure of southern Africa populations. (A) Southern Africa populations analyzed in this study. Different Colours are associated with different language/ethnic affiliation. The complete dataset used for analysis is shown in Figure S1 and Table S1. (B) Admixture results for K=10,15,20 (from the inner to the outer circle). Colours at the center reflect the affiliation shown at (A) and Figure S1. We analyzed 1856 individuals for 91 populations, and averaged the results in a population based barplot. The full set of results (K={1..20}) for individuals and populations is reported in Figure S2 and Figure S3.

Our analyses focus on southern African populations. We therefore merged our data with an additional 31 Khoesan-speaking, and 20 “admixed” and Bantu-speaking populations (Li *et al.* 2008; International HapMap 3 Consortium 2010; Henn *et al.* 2012; Pickrell *et al.* 2012, 2014; Schlebusch *et al.* 2012; Petersen *et al.* 2013; Lazaridis *et al.* 2014) (Figure 1, Supplemental Material, Figure S1, Table S1, and File S1). Additional data from outside of southern Africa were taken from populations with European, African, and Middle East ancestry, genotyped on different Illumina platforms and the Affymetrix Axiom Genome-Wide Human Origins 1 array (Figure 1, Figure S1, and Table S1) (Li *et al.* 2008; International HapMap 3 Consortium 2010; The 1000 Genomes Project Consortium 2012; Patterson *et al.* 2012; May *et al.* 2013). Our final dataset comprised 1856 individuals from 91 populations (Figure 1 and Table S1).

### Process for merging datasets

Because the genotype data described above came from multiple different platforms and studies, we performed a systematic pipeline for merging the data, keeping the Illumina and Affymetrix data initially separated. Each dataset was preprocessed, removing markers and individuals with a missing rate higher than 10%, using PLINK 1.9 (Chang *et al.* 2015). Marker positions were lifted to build 37 human genetic maps using data provided by Illumina and Affymetrix, and all nonautosomal markers were excluded from the analysis. Specifically, we first merged all the datasets genotyped on the same platform, discarding individuals and markers with a call rate lower than 98%, and excluding SNPs with G/C or A/T mutations, which could lead to errors in the merging procedure. Although, in principle, merging genotype data from different platform manufacturers could led to errors or biases, this approach has been employed successfully in previous investigations (Reich *et al.* 2009; Henn *et al.* 2012). In addition, in none of the analyses performed we report differences between related groups genotyped with different platforms. Moreover, the identity-by-state (IBS) similarity between 328 pairs of individuals that have been genotyped by the two manufacturers was always higher than 0.996 (99% C.I.=0.998−1.000). We next used the KING software to infer kinship (Manichaikul *et al.* 2010), and randomly removed one individual from pairs with a kinship rate higher than 0.0884. The resulting platform-specific datasets comprise 250,547 (Illumina) and 498,140 (Affymetrix) markers, respectively.

### The unphased dataset

To maximize the number of populations analyzed (at the expense of SNP density) we merged all data collected from all studies into a large dataset, which we refer to as the “Unphased dataset”. The two platform-specific datasets, comprising 250,547 (Illumina) and 498,140 (Affymetrix) markers, were merged on physical position to avoid unnecessary loss of markers due to mismatching IDs on different platforms. Following this merge, we again performed the same quality control and removal of relatives described above, obtaining a final dataset containing 1856 individuals genotyped at 63,767 SNPs.

### The Khosean ancestry dataset

To maintain a high density of SNPs for local ancestry analyses, we analyzed the quality controlled Illumina and Affymetrix datasets separately. For each of the two platform-based datasets described above, we computationally phased the genotype data to generate haplotypes using SHAPEITv2 (Delaneau *et al.* 2012, 2013) with the human genome build 37 recombination map downloaded from the SHAPEIT website (https://mathgen.stats.ox.ac.uk/genetics_software/shapeit/shapeit.html#gmap). We generated a second dataset (“The Khoesan Ancestry dataset”), by initially removing from each platform-specific dataset (Illumina and Affymetrix) non-Khoesan genomic fragments as identified using PCAdmix (Henn *et al.* 2012). In brief, PCAdmix builds a principal components analysis (PCA) space based on reference panels, and projects tested genomic chunks on it; similar approaches have been previously developed (Price *et al.* 2009; Omberg *et al.* 2012; Maples *et al.* 2013). Subsequently, the probability of a given ancestry for a selected chromosomal chunk is estimated from principal component (PC) loadings and a hidden Markov model is then applied to refine them. In the current context, we estimated local ancestry likelihoods in 1 cM windows, using Yoruba, Ju|’Hoan, and CEU individuals as ancestry donors. Given the recent West Eurasian genomic component documented in the Ju|’Hoan populations as the result of admixture with non-Khoesan populations (Pickrell and Pritchard 2012; Hellenthal *et al.* 2014; Busby *et al.* 2016), only individuals with >99% of the “Khoesan component”—as estimated by the K=3 ADMIXTURE run described below—were considered as donors, with the remaining individuals used as target individuals. The final number of Ju|’Hoan individuals used as ancestry donors was 28 and 26 in the “Illumina Local” and “Affymetrix Local” datasets, respectively. To minimize the impact of chunks with mixed ancestry, we postprocessed inferred local ancestry estimates by retaining only those windows with a ancestry probability >99%. In addition, we only analyzed individuals characterized by >20% of the tested ancestry (as for ADMIXTURE analysis for K=3; see below). We tested the accuracy of PCAdmix on the Illumina dataset using a simple approach. In detail, using the same source populations (Yoruba, Jux’hoan, and CEU) and parameters, we estimated the local ancestry of 73 Yoruba individuals. When no threshold confidence was used, the 0.8% of the analyzed 1 cM windows were misassigned. However, when only windows assigned with >99% confidence were retained, all the misassigned fragments were discarded.

We used a custom-made PYTHON script (*MaskMix*, available at: https://capelligroup.wordpress.com/tools/), to extract the Khoesan Specific Fragments (KSF) inferred from the postprocessing described above. *MaskMix* considers each individual as homozygous, and composed by one chromosome per pair only, from which high confidence KSFs were extracted and analyzed. This approach allows us to use chromosomal data instead of individual genotypes, maximizing the amount of genetic data suitable for the analysis, and is not expected to affect any of the analyses performed because the relative allele frequency would be unchanged. To allow the comparison between individuals genotyped using arrays from different providers, the resulting two datasets were pruned to retain markers that overlapped between the Illumina and Affymetrix datasets and that were located on Khoesan-specific genomic fragments. Finally, we removed all the individuals for which <10% of the total number of overlapping SNPs were retained. The resulting dataset is composed of a total of 63,767 markers and 787 individuals. Given the variation in Khoesan ancestry in different individuals, the average number of retained SNPs per individual was 22,442 (median 19,887; range 5457–50,643). We refer to this final set of SNPs selected as described above as the “Khoesan Ancestry dataset”. Furthermore, we assessed the different performance of the two datasets (Illumina and Affymetrix), exploring the distribution of the tract length estimated by the approach with respect of the platform manufacturer (Figure S2).

### Statistical analyses

#### Population structure:

**Figure 2:**
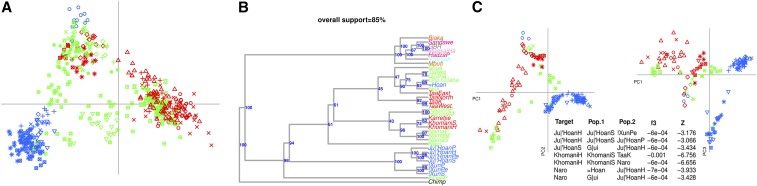
Local ancestry deconvolution reveals complex Khoesan-related structure. (A) MDS of Khoesan specific fragments. We extracted fragments with high (>99%) probability to be derived from Khoesan populations, and visualized it in a MDS plot, as described in the section *Materials and Methods*. (B) ML tree of Khoesan populations. We selected all the Khoesan populations, and added seven African and European populations. We performed 10 different runs and assessed the support of each tree through 100 bootstraps (Figure S6). Colour keys are as in Figure 1A and Figure S1. (C) PCA of individuals with >80% of Khoesan-related genetic ancestry. We used the *K* = 3 ADMIXTURE run to select individuals characterized by at least 80% of Khoesan genetic ancestry, and performed a PCA as described in the section, *Materials and Methods*. The two most significant $f_{3}$ between “Target” and sources (“Pop.1” and “Pop.2”) populations, including SD and *Z*-score, are reported.

We applied both model-based and nonparametric clustering approaches to describe population structure in the Unphased Dataset. First, we used the ADMIXTURE (Alexander *et al.* 2009) maximum likelihood (ML) algorithm to estimate the individual-level ancestry, applying the author’s cross-validation procedure and a random seed, for all values of K={2…20}. Ten different runs for each value of *K* were performed, and different outputs were combined using the CLUMMP utility in CLUMPAK (Jakobsson and Rosenberg 2007; Kopelman *et al.* 2015) with the LargeKGreedy algorithm, random input order, and 2000 repeats. After postprocessing the ADMIXTURE output with DISTRUCT (Rosenberg 2004), we plotted the results using the *R* statistical programming software, and a modified version of *polarHistogram* function from the *phenotypic phorest* package (http://chrisladroue.com/phorest/). For the K=20 run with the highest likelihood value, we computed pairwise $F_{ST}$ (Holsinger and Weir 2009) for each of the *K* ancestral components as implemented by ADMIXTURE, visualizing their distances with a heatmap using the *pheatmap* (Kolde 2015) *R* package. Ancestral components were additionally clustered through a complete hierarchical approach (Everitt and Britain 1980). For the K=20 analysis with the highest likelihood ratio, we estimated splitting time between the three components and all the Khoesan populations using the following formula (Holsinger and Weir 2009; Henn *et al.* 2012): $1-F_{ST}={\left(1-\frac{1}{2Ne}\right)}^{t}$ where *Ne* is the effective populations size, and *t* is the time since separation (in generations). This approach has been applied to pairwise $F_{ST}$ among admixture ancestries and populations, although in the latter we removed individuals characterized by <80% Khoesan ancestry, as reported below. We used the *Ne* inferred over time for five Khoesan by Kim *et al.* (2014), from which we extracted the armonic mean, the mean value, and the final population size estimate (10,024, 12,302, and 14,024). The density of the splitting times between the three ancestries using the two approaches is shown in Figure S3. PCA was performed using PLINK 1.9 (Chang *et al.* 2015). To focus on the structure of Khoesan populations, we selected only those individuals characterized by >80% of the “Khoesan” ancestral component as estimated from the K=3 ADMIXTURE analysis described above; we define the “Khoesan” component as the major ancestry present in Jux’Hoan individuals. We refer to this dataset as the “80% Khoesan dataset.” Admixture between populations was assessed using $f_{3}$ statistics. (Reich *et al.* 2010; Patterson *et al.* 2012) considering all three-populations combinations (3990 combinations, Table S3). We report all the comparisons in Table S3, while significant values are reported in Figure 2C. In addition, we performed two different set of $f_{4}$ analysis, using the qpDstat software, and the option “f4mode = YES”. In details we performed the $f_{4}$ stat in the form Ju|′hoanPe,Nama,X,Chimp), where *X* is represented by all the other populations in the unphased dataset (Figure S4A). In order to remove the effect of recent admixture, we repeated the same test on the “Khoesan Ancestry” dataset, the Illumina Khoesan Ancestry (“Illumina Local”) and the Affymetrix Khoesan Ancestry datasets (“Affy Local”). Moreover, we assessed the *f_4_* test of the form $f_{4}\left(Khoesan1,Khoesan2,X,Chimp\right),$ where *X* is one of the admixed (Basters and Coloured) or Southeast Bantu populations (Figure S4C).

#### Evaluating ADMIXTURE performance on simulated samples:

As we describe in the results, at K=14, ADMIXTURE identifies an *European* ancestral component that characterizes the admixed populations (Coloured and Basters). However, the $F_{ST}$ distance between this component and the African ones (Figure S5) is smaller than the distance between the African and the other European ancestries, which could suggest that the algorithm picked a new combination of allele frequencies caused by the admixture. To test this hypothesis, we evaluated the performance of ADMIXTURE on simulated admixed samples. In details, we used the Yoruba (YRI) and British (GBR) genetic data and generated four groups of 25 admixed individuals composed by a variable fraction (20%−40%−60%−80%) of British and Yoruba individuals, which admixed *N* generations ago (N=5,10,30,50,70,and 100). The admixed individuals have been combined with the sources groups, and the final datasets have been used to perform an ADMIXTURE run for K=2 and K=3 (Figure S6).

#### TREEMIX analysis:

A ML tree describing the relationships between Khoesan populations was inferred using allele frequency distributions implemented in the TREEMIX software (Pickrell and Pritchard 2012; Pickrell *et al.* 2012). Given the high complexity of the original dataset, we selected 35 populations to represent all the Khoesan populations, and included a subset of African and European populations (“TreeMix analysis,” Table S1). We used a chimpanzee outgroup using genome data available in Patterson *et al.* (2012), and accounted for linkage disequilibrium (LD) by jack-knifing over blocks of 500 SNPs, as suggested by the authors in Pickrell and Pritchard (2012). The robustness of the resulting tree was tested by performing 100 bootstrap runs, and estimating branch support using DENDROPY software (Sukumaran and Holder 2010).

We performed 10 different runs using different random seeds (Figure S7), and report the tree with the maximum support in Figure 2B. To visualize only the Khoesan ancestry and remove the confounding factors due to admixture, we informed TREEMIX of existing relationships between Khoesan and non-Khoesan populations using the cor_mig and climb commands (Table S2), as estimated by the K=3 ADMIXTURE run described above. It is important to note that these estimates are not fixed values, but are used by the algorithm as starting points to infer the ML estimates (Pickrell *et al.* 2012).

#### Population structure inference using the Khosean Ancestry dataset:

**Figure 3:**
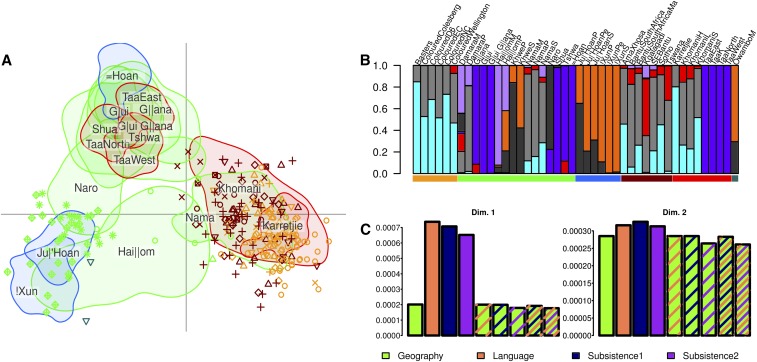
(A) Genetic structure of admixed southern African populations. In order to provide a simplified version of Figure 2A, we estimated the 90% utilization kernel of Khoesan populations (except Damara and Khwe, see text), and plotted the highly admixed individuals. (B) Cluster analysis of genomic fragments. We grouped all the individuals in seven clusters, as inferred by Mclust *R* package (see *Materials and Methods*), and visualized the results in barplots according to populations and language/ethnic affiliation. Colour keys are as in Figure 1A and Figure S1. The results highlight the large heterogeneity in populations sharing the same affiliation, and the existence of a slight but significant substructure between Bantu and Coloured populations. (C) Predictive errors of genetic components for geographic, linguistic, and subsistence affiliation, or a combination of different covariates (striped bars), for the first two dimensions of the MDS in Figure 2 and (A) (Dim 1 and Dim 2, respectively). Geography better predicts genetic ancestries, though adding new covariates slightly decreases the predictive error.

Referring to the Khosean Ancestry dataset, we estimated pairwise (1-IBS) genetic distance with PLINK 1.9, correcting for missing data, and summarized relationships with a Local Ancestry Multi-Dimensional Scaling (LAMDS) plot, using the *cmdscale* function in *R* (Figure 2A and Figure 3A). We corrected the inferred IBS-based distances using the formula ibs/1−∑md, where md is the number of missing data in the pair of individuals analyzed. Furthermore, after visual inspection, we removed 25 outlier chromosomes (one chromosome each from AmaXhosa, Basters, ColouredEC, colouredWellington, and Khwe; two from SEBantu and Sotho; three from Tswana and Kgalagadi, eight from BantuSouthAfricaMa). Distances were computed using the number of SNPs shared across pairs of individuals, which differs across pairs given the variation across individuals in the number of SNP markers found on Khoesan genomic fragments. The average number of markers used for individual to individual comparisons is 9310 (median = 6404; range = 111–46,419). In order to assess the bias that a small number of SNPs may cause in capturing the genetic variation in the area, we resampled 10 different datasets composed by *N* markers, with N={500...1000…10,000} and compared the median and 95% C.I. with the whole dataset. In addition, we reported the average $R^{2}$ between the resampled and the full datasets.

#### Structure and distribution of Khoesan ancestry in southern African populations:

To assess the presence of ancient structure in Khoesan speaking populations, and the existence of different Khoesan ancestry in admixed Bantu and Coloured individuals, we explored the multidimensional scaling (MDS) coordinates and IBS distance matrix. We initially plotted all southern African groups with 90% utilization distribution density kernels for the three ancestries, estimated using the Kernel Utilization Distribution (UD) function in the *adehabitatHR* *R* package (Calenge 2006). The function estimates the minimum area of the plot in which individuals from the same population have the 90% probability of being located. First, we used the MDS coordinates and IBS distances to group all individuals into *N* different clusters, for values of N={1…9}, using the algorithm implemented in *Mclust* *R* package (Fraley and Raftery 2002). We next visualized the average assignment probabilities into population- and affiliation-based barplots. Finally, to test for a correlation between genetic and geographic distances, we performed a Procrustes test (Peres-Neto and Jackson 2001) as implemented in the *R* package *ade4* (Dray and Dufour 2007; Dray *et al.* 2007), with 1000 bootstrap iterations between geographic and MDS (genetic) coordinates.

#### SpaceMix analysis:

In order to further investigate the distribution of the genetic variability among the three Khoesan ancestries, we built a “GeoGenetic map” of populations (only individuals with >80% Khoesan ancestry), taking advantage of the Bayesian statistical framework implemented in SpaceMix (Bradburd *et al.* 2016). Briefly, this approach reconstructs the genetic relatedness among populations as a map in which distances are proportional to their genetic dissimilarities. Moreover, inferred long distance relatedness are modeled as gene flow between populations. In detail, we ran an analysis as in Bradburd *et al.* (2016): first, five independent short chains of $5*10^{6}$ Markov Chain Monte Carlo interactions, in which only locations were estimated. For the whole analysis, the initial population locations were taken by a uniform distribution with minimum and maximum of -180, 180 and -90, 90 longitude and latitude, respectively. Second, a long chain of $10^{8}$ iterations sampled every $10^{5}$ steps was analyzed. The starting parameters of this chain were taken by the last iteration of the short run characterized by the highest posterior probability. Finally, the inferred “geogenetic position” (and their 95% C.I. ellipses) and their sources of admixture were superimposed on observed population sampling locations (Figure S8A). The overall performance of the approach was assessed by exploring the posterior probability trace (Figure S8B), while the ability of the model to describe the data was evaluated by analyzing the correlation between parametric *vs.* observed covariance matrix (Figure S8, C and D) and the decay of covariance *vs.* geographic distance for observed and inferred matrices.

#### Estimating predicting power for geographic location, linguistic affiliation and type of subsistence:

To assess the role of geography, subsistence, and language in predicting genetic variation of Khoesan populations, we performed regression analysis of LAMDS first two components *vs.* all the other variables, singularly or combined (“Geography + Language”, “Geography + Subsistence,” and “Geography + Language + Subsistence”; Table S4). All the combinations were tested through a five-fold cross-validation analysis in which the dataset was split into five random subsets. Each of these subsets was then tested against the other four, and the combined error recorded and shown in a barplot (Figure 3C). Two alternative subsistence affiliation lists were used to take into account uncertainty in designation, or the coexistence of multiple subsistence strategies. In detail, “Subsistence 1” was annotated according to Schlebusch *et al.* (2012) and Barnard (1992). In order to take into account multiple, and/or uncertain, subsistences in Damara and admixed populations, we used an alternative list (“Subsistence 2”), in which these groups were indicated as Hunter-Gatherer/Herder and farmers, respectively (Table S4).

#### Estimating admixture dates using MALDER:

We assessed possible evidence for admixture using the algorithm implemented in MALDER (Pickrell *et al.* 2014), which, developed from ALDER, fits a mixture of exponential functions to weighted LD density curves, allowing multiple admixture events to be identified. We performed a MALDER analysis for all the populations with more than two individuals using the “mindis:0:005” parameter. The results of our analysis are shown in Figure S13, in which we report the estimated dates (±1 SD), and the two populations generating the highest amplitude for each inferred event. In addition, for each event, we assessed if the other amplitude estimates were significantly different than the maximum one (Z>2). *Z* was estimated using the formula:

$$Z=\frac{C_{\text{max}}-C_{\text{nonmax}}}{\sqrt{se\left(C_{\text{max}}^{2}\right)+se\left(C_{\text{nonmax}}^{2}\right)}}$$

We classified the two populations that generate nonsignificantly different $Z_{\text{score}}$ (Z<2) into one of the macrogroups shown in Figure 1A. Next, for each admixture event, we showed the frequency of comparisons in which a group is inferred as source. Each dataset from a different platform manufacturer was analyzed individually. Although the overall interpretation of the results can still be challenging, especially because of the different size of macro-groups, it could provide additional insights on the likely real source.

### Data availability

All the new genotypes presented here are available for download at https://capelligroup.wordpress.com/data/. The MaskMix utility program is available at https://capelligroup.wordpress.com/tools/.

## Results

### Complex population structure and mixed ancestry in southern Africa

We describe population structure in southern Africa using the “Unphased Dataset” (see *Materials and Methods*) comprising 1856 individuals from 91 populations genotyped at 63,767 autosomal markers (Figure 1 and Figure S1). After kinship analysis, 25 individuals were removed, one from each of the 25 inferred pairs of highly related individuals. Ten of these pairs contained individuals genotyped in different studies: two Bantu South Africa from the HGDP (Li *et al.* 2008) with a high kinship index with two Herero from Schlebusch *et al.* (2012), three and five pairs among the Ju|’Hoan [from Pickrell and Pritchard (2012), Schlebusch *et al.* (2012), and Petersen *et al.* (2013)], and the Khomani [from Henn *et al.* (2011) and Schlebusch *et al.* (2012)], respectively.

To visualize the evolutionary relationships among the analyzed individuals, we used ADMIXTURE (Alexander *et al.* 2009), varying the prescribed number of clusters, *K*, from 2 to 20 (Figure 1B, Figure S9, and Figure S10). At K=7, all African populations are mostly characterized as a mixture of four African-specific components defined by language, geography, or ethnicity, representing Khoesan (red and blue), Niger-Congo (turquoise), East African (purple), and rainforest Hunter-Gatherer (Pygmies, orange in Figure 1) populations. Interestingly, the latter is also present in Western and Eastern Bantu populations, and in the Hadza, Sandawe, and Maasai from East Africa, possibly reflecting admixture, and/or the existence of a geographically extended Pygmy-related ancestral component (Destro-Bisol *et al.* 2004; Tishkoff *et al.* 2009; Patin *et al.* 2014).

Among Khoesan groups, signatures of admixture and possible cultural transition are evident in most of the populations. For example the Damara show a high fraction of Bantu-like ancestry components (Figure 1B, Figure S9, and Figure S10). More generally, almost all of the Khoesan populations show a non-negligible fraction of ancestry components that are modal in East Africa and Europe, consistent with ancient and recent migrations from these regions (Tishkoff *et al.* 2009; Schlebusch *et al.* 2012; Pickrell *et al.* 2014). At K=8−10 additional Eurasian components emerge, which differentiate from Afro-Asiatic ancestries.

We noted that, although the smallest estimated cross-validation values are found for K=9 and 10 (Figure S11), analyses performed at higher values of *K* provide insights into the genomic history and substructure of populations, so we describe these results below. At K=12, the component common in Niger-Congo speaking populations splits into two, one common in Western and Central African populations and in Western Bantu speakers, the other more common in Southeastern Bantu speakers, consistent with these representing the last stage of the Bantu migration process (González-Santos *et al.* 2015). Interestingly, this Southeastern Bantu component is present in most Khoesan populations from Botswana, Lesotho, and South Africa, providing evidence for admixture during the expansion of Bantu-speaking populations (Tishkoff *et al.* 2009; Pickrell and Pritchard 2012; Schlebusch *et al.* 2012; Barbieri *et al.* 2013b; Petersen *et al.* 2013; González-Santos *et al.* 2015; Marks *et al.* 2015; Busby *et al.* 2016). The presence of ancestry related to Western Bantu speakers in some of the Western Khoesan populations such as the Khoe, the Hai||om, and the Nama, is consistent with their current geographic position, and could be interpreted as a signature of admixture events. At K=13, a component which almost exclusively characterizes Western Bantu populations becomes evident. Interestingly, this component is modal in the Damara, Herero, and Himba populations, providing some evidence for a closer affinity (Barbieri *et al.* 2013a). In addition, this same ancestral component is at high frequency in all the other Bantu populations, where it is complemented by the presence of the Southeastern Bantu component. At K=14, an ancestry present only in the recently admixed population of South Africa and Namibia (Coloured and Basters) emerges: $F_{ST}$ values of this component suggests genetic similarity with European populations. However, the genetic distance between this ancestry and the other African components is consistently smaller than the values estimated when using European populations (Figure S5), which suggests that the admixture between African and Eurasian populations might have generated a novel combination of allele frequencies, which is now captured by this component (Figure 1B, Figure S9, and Figure S10). We tested this hypothesis by exploring admixture runs performed on different simulated populations composed by a variable fraction of British (GBR) and Yoruba (YRI) ancestry. In all the simulations, the ADMIXTURE software models a third component (K=3),which is characterized by a mixture of the other two (Figure S6). At K=15, the Sandawe population differentiates from other groups from Tanzania, Hadza, and Maasai.

### Local ancestry analysis reveals three distinct Khoesan-related ancestries

Ancestry analysis of Khoesan populations is complicated by the fact that the genomes of most of the contemporary populations are a mosaic of multiple ancestries (Pickrell *et al.* 2014; Marks *et al.* 2015; Busby *et al.* 2016). For this reason, we performed a LAMDS analysis using only genomic fragments assigned with high confidence to Khoesan ancestry (“Khoesan Ancestry dataset,” see *Materials and Methods*). Similar methods have previously been successful in assessing the continental legacy of American populations (Moreno-Estrada *et al.* 2013). However, such methods rely on a large set of reference populations that can be used as a scaffold for local PCA visualization, which were not available here. We therefore applied a new LAMDS approach, in which an IBS-based distance matrix was generated by comparing only those variants on chromosomal segments identified as being of Khoesan ancestry. This analysis has the advantage of using chromosomes instead of individuals, and allows one to plot admixed populations even when there is not a comprehensive reference dataset. We assessed the possible impact of variable number of markers analyzed through a resampling procedure (see *Materials and Methods*). The mean correlation of IBS between the whole and the resampled dataset is always higher than 0.8, and reaches 0.9 with as little as 1500 markers, which suggests that the impact of different numbers of markers on IBS estimates is negligible (Figure S12). In addition, the tract length density for the two datasets is very similar (Figure S2), with the number of short chunks identified for the Affymetrix dataset being slightly larger than the Illumina. However, given that missing data are excluded in our pairwise IBS distance estimation, we do not expect this to cause substantial bias. Our LAMDS analysis reveals three main groups of Khoesan-related ancestry (Figure 2A). The first group (*Northern* Khoesan) is composed of all the K’xa speaking populations located at the North of the Kalahari (Ju|’Hoan and !Xun) with the exception of the more central =Hoan. The other two groups are composed by the Nama, Khomani and Karretjie (*Southern* Khoesan), and all of the remaining Khoesan populations (*Central* Khoesan).

Next, we investigated the presence of three-way Khoesan genetic structure with a series of analyses using the “Unphased dataset.” First, we built ML trees from allele frequencies using TREEMIX, and tested their robustness with bootstrapping. Khoesan populations [with the exception of the Damara, who were excluded from this analysis due to their low Khoesan ancestry (Figure 1B)] form three groups, a pattern consistently found across bootstraps. This three-way partition broadly mirrors the clustering patterns of the LAMDS analysis (Figure 2B and Figure S7). The Karretjie appear more related to the Khomani than to the Nama, with the Naro acting as an outgroup to these two branches. Similarly, Hai||om and Shua form a distinct branch that splits from the other Southern Khoesan, although there is little support for this branching pattern across bootstrap runs. The split, which separates the !Xun from the JulHoan, is well supported, further emphasizing the genetic distinctiveness of these two K’xa populations (Petersen *et al.* 2013).

PCA using individuals with at least 80% Khoesan ancestry provided additional evidence for the Khoesan ancestry tripartition (Figure 2C). Specifically, the three vertices of the plot recapitulate the ADMIXTURE and TREEMIX analyses, with the three clusters composed by populations with different amounts of *Northern*, *Central*, and *Southern* Khoesan ancestries. We note that, in the ADMIXTURE analysis described above, at K=16 three Khoesan-related components emerge, separating all the populations from the central Kalahari area (Botswana) speaking Taa, K’xa, and Khoe-Kwadi (*Central* Khoesan) from the K’xa in the North (*Northern* Khoesan) and the Nama, Khomani, and Karretije in the South (*Southern* Khoesan). Notably, the $F_{ST}$ values between these three components are similar, suggesting either a deep split (possibly followed by admixture), and/or drastic demographic events, such as bottlenecks or founder effects. Using $F_{\text{ST}}$ and sample sizes for Ju|’Hoan and Taa (Kim *et al.* 2014), we estimate a splitting time of ∼25 KYA (95% C.I. 18–32 KYA among all $F_{ST}$ and sample size combinations, Figure S3A) when ancestral components inferred by ADMIXTURE are used, which is broadly consistent with previous estimates (Pickrell *et al.* 2012; Kim *et al.* 2014). When we use pairwise population $F_{ST}$ values (Figure S3B), and assume a generation time of 29 years (Fenner 2005), the inferred split time is 14 KYA (2–27 KYA), likely reflecting the effect of admixture involving Khoesan and/or non-Khoesan populations. Nevertheless, both approaches seem to demonstrate that this genetic structure could have a prehistorical rather than historical origin. Among the Basters and the Coloured, the *Southern* Khoesan component represents most of the Khoesan-like ancestry, while, conversely, in South African and Lesotho Bantu-speaking populations, the major component is *Central* Khoesan. Furthermore, a substantial number of Khoesan populations show a combination of these three ancestries, suggesting extensive admixture in the history of these populations.

Interestingly, five out of the 11 of the *Central* Khoesan populations are closer to *Northern* populations by means of the $f_{4}\left(Ju|hoanPe,Nama,X,Chimp\right)$ test, suggesting gene flow between these two groups (Figure S4). However, interpretation of these tests is challenged by admixture events between nearby populations, which skewed the allele frequency of the “source” populations. When the same test is performed only on “Khoesan fragments,” this signature is retained only for the Naro population (Figure S4), which behaved as truly admixed in multiple analyses. Although a low degree of admixture between *Central* and *Southern* groups cannot be excluded, this is unlikely to explain the observed genetic structure. On the contrary, the results are compatible with a isolation-by-distance scenario (see also SpaceMix results below).

We used MALDER to provide a temporal dimension to the observed admixture events, a method that exploits LD decay to infer the time of admixture between populations (Figure S13). Overall, the results are consistent with earlier work (Pickrell and Pritchard 2012; Pickrell *et al.* 2014; Busby *et al.* 2016). Among Khoesan populations, many have signatures suggesting two different admixture events; the first, <10 generations ago and involving African and non-African populations, is concordant with colonial times in the region, while the second, involving similar sets of populations ∼40−60 generations ago (∼1160−1740 years ago), is probably related to the arrival of the pastoralists in the area (Pickrell *et al.* 2012, 2014). All the Coloured populations share with the Khoesan recent episodes of admixture (∼4−7 generations ago, ∼116−203 years ago), with the exception of BantuSouthAfricaMa. The Southern-east Bantu share an earlier admixture event dated ∼17−32 generations ago (∼493−923 years ago) consistent with the arrival of Bantu speaking populations in the area. The BantuSouthAfricaMa show evidence of five admixture events, which could be explained by its heterogenous composition (May *et al.* 2013).

We modeled the geography of population structure in Khoesan populations, taking advantage of the Bayesian statistical framework implemented in SpaceMix. The resulting geogenetic map, which summarizes the genetic structure and the admixture events among populations, is shown in Figure S8A. The results are consistent with our previous analyses. For example, the 95% ellipses in the geogenetic map highlight the existence of three main Khoesan groups (Figure S8A). This approach detects apparent substructure within the three clusters, such as the genetic differentiation between !Xun and Ju|’Hoan, or between Khomani and Nama. The *Central* group seems to be further subdivided into a Eastern and Western group. Several admixture events (α>1%) were identified, confirming the existence of past relationships between the three groups. We inferred large non-Khosean contributions in the Khomani, !Xun, Jux’Hoan and Naro, among several other populations. The run tracts and the correlation among the inferred and observed parameters suggest that the analysis and the model accurately describes the observed data (Figure S8, B–D).

### Contemporary Khoesan populations contain a mixture of Khoesan-related ancestries

Our LAMDS analysis offers further insight into the relationships between Khoesan groups (Figure 2A). In fact, chromosomes from several populations seem to be scattered between different clusters, potentially as a result of admixture. For example, Khomani individuals are spread toward groups enriched in *Central* Khoesan ancestry, the Naro and some of the Jux’Hoan occupy a position intermediate between populations characterized mostly by *Central* and *Northern* components and the Hai||om are scattered between individuals with *Northern* and *Southern* Khoesan genetic profiles. These results are consistent with our PCA analyses based on the subset of individuals in each group with >80% Khoesan ancestry. It is important to note that all the analysis converge toward a tripartite genetic structure in Southern Africa, suggesting that the error due to the “computational phasing” is negligible. In addition, it has been shown previously (Hellenthal *et al.* 2014) that different phasing methods tend to generate consistent results.

PCA confirms patterns similar to those described above for the Khomani and Naro, which are spread toward groups rich in *Central* and *Northern* Khoesan ancestry, respectively (Figure 2C). We formally tested for admixture between populations, applying the $f_{3}$ analysis on the same dataset (Figure 2C); among the significant tests, we reported the two most negative *Z*-scores for each population tested; all the comparisons are reported in Table S3. Significant $f_{3}$ statistics provide evidence that these mixed ancestries are the result of admixture between different Khoesan populations (Figure 2C and Table S3). None of the *Central* Khoesan populations show significant evidence of admixture between *Northern* and *Southern* groups. In the Khomani, the lowest $f_{3}$ values are found when considering Taa populations (Figure 2C and Table S3). The Naro show evidence of admixture involving populations close to Ju|’Hoan and a central Khoesan population, such as Taa and G|ui. The Ju|’Hoan also show significant $f_{3}$ values when tested against !Xun (*Northern* Khoesan) and Naro (*Central* Khoesan). Similarly, the !Xun also show evidence for admixture with the Ju|’Hoan.

### Khoesan-related genetic structure in admixed populations

To better visualize the relationships between populations in relation to their Khoesan ancestries, we initially plotted all southern African Khoesan (with the exception of the Khwe and Damara) groups with 90% utilization distribution density kernels for the three ancestries (Figure 3A), estimated using the KernelUD function in the *adehabitatHR* package (Calenge 2006). Next, we added the remaining admixed population points. This approach allows us to explore which of the three ancestral components is present in Bantu-speaking, admixed, and Khoesan groups with high Bantu ancestry, such as the Khwe and the Damara. The Khwe cluster with the sympatric Ju|’Hoan and !Xun populations, although some individuals are located closer to populations mostly containing a *Central* Khoesan component, potentially reflecting a non-negligible degree of admixture. The Damara, conversely, seem to be genetically closer to the Khomani and Nama, although they are scattered toward the K’xa populations in the North, in accordance with their geographic location. Interestingly, we identified two Owambo individuals with genomic features related to Ju|’Hoan and !Xun populations.

All of the other Bantu-speaking groups—with the exception of the Kwangali, who are closer to Taa and K’xa speaking groups from Botswana (*Central* Khoesan)—are genetically related to the cluster defined by Nama, Karretjie, and Khomani (*Southern* Khoesan). We noted that admixed individuals mapping to this cluster appear to highlight a partly structured distribution, since the Bantu populations are located on the upper side of the distribution, while Coloured and Basters are on the lower side (Figure 3A). To test this hypothesis, we used *mclust* to explore the most supported number of clusters, from one to nine inclusive, using either the MDS coordinates or the IBS distance matrix. Using the Expectation-Maximization model based clustering algorithm, we inferred seven clusters using the MDS coordinates, and nine with the distance matrix; the average probabilities for each population are shown in Figure 3B and Figure S14. In both analyses, Coloured and Bantu populations are defined mostly by the same cluster affiliation, although present in different proportions. An additional minor cluster, related to Bantu-speaking populations from Botswana, is present in the Southern Bantu populations, but absent in the Coloured and Basters. Such differences are still evident when the complete distance matrix is considered (Figure S14). These results are supported by the test $f_{4}\left(Khoesan1,Khoesan2,X,Chimp\right)$ which shows a marked difference between Bantu and Coloured populations. In detail, all the Bantus show affinity with “Central” (G|ui G||ana individuals) or “Southern” Khoesan (Nama) when tested against the Northern-panel, while Basters and Coloured only with Southern Khoesan (Figure S4C). However, caution must be used in the interpretation of these tests when admixed populations are used as “Sources.”

### Khoesan-related genetic structure and geography, language and subsistence

We performed a Procrustes analysis to test the relationship between genetic and geographic distances, and found a statistically significant correlation (Procrustes correlation = 0.65, *P* < 0.001), as previously observed by Schlebusch *et al.* (2012), across a small subset of Khoesan populations. Here, we extended this analysis to include not only a larger dataset of populations, but also Khoesan fragments in highly admixed groups such as southern-African Bantu-speaking populations, and Coloured. To further investigate the association between geography and the observed Khoesan-related structure, and to explore the correlation with cultural variables, we evaluated the power of models predicting the positioning of individuals along the two dimensions of the Khoesan-ancestry IBS-based MDS plot (Figure 3C) for geography, language, and subsistence. Major reductions in model predictive error, which is indicative of better model fit, are observed only when variables are considered in relation to MDS dimension 1 (Figure 3C), while dimension 2 shows some degree of model prediction reduction only when geography is considered (Figure 3C). Geography shows the smallest predictive error, and therefore best model-fit, when each variable is singularly considered (geography: 0.000201, language: 0.0007, subsistence1: 0.0007, subsistence2: 0.000652). Although the predictive power of the analysis is improved when multiple variable are considered, the reduction of cross-validation error is minimal (Geography + Language: 0.0002, Geography + Subsistence1: 0.000199, Geography + Subsistence2: 0.000179, Geography + Language + Subsistence1: 0.000192, Geography + Language + Subsistence2: 0.000177). Overall, the observed Khoesan ancestry in well represented by the geographical distributions of populations. Such genetic structure likely predates the arrival of Bantu and European populations, and is only marginally captured by current ethno-linguistic population descriptors.

## Discussion

The genetic characterization of populations from the African continent is crucial from an epidemiological, pharmacological, anthropological, and evolutionary perspective. Within the continent, southern Africa displays an impressive degree of genetic and cultural diversity, this being a region where groups speak several languages, and implement a variety of different strategies. From a linguistic point of view, Khoesan languages are unique to this region, and are classified into three major families: K’xa, Khoe-Kwadi, and Taa. While the separate grouping of K’xa and Taa speakers has reached a consensus among linguists, the internal structure of the Khoe-Kwadi family is still debated. The most heterogeneous of the three linguistic groups, Khoe-Kwadi, is usually classified into three subgroups; East (spoken by Thswa and Shua), West (Khwe, G|ui, G||ana, and Naro), and Khoekhoe, which is currently spoken by the Nama, Damara, and Haixxom populations (Guldemann and Fehn 2014). The history of Khoekhoe populations still remains unresolved; for example, the Hai||om and Damara have previously been classified as “other bushmen” when their phenotypic, linguistic, and/or cultural characteristics were considered (Barnard 1992). The Hai||om live in Northern Namibia, and they are thought to be !Xun individuals that have recently acquired the Nama language. The Damara—who were sometimes referred to as BergDama or BergDamara—live in Northern Namibia, and their origins are also unclear. Including both herders and foragers, the ancestral population probably arrived in the area before the Nama and Western Bantu populations, such as Herero and Owambo. The arrival of the Nama pastoralists in the Namibia region from an area in the South African Northern Cape (Namaqualand) is a recent event dating to the end of the 19th century (Barnard 1992). The first pastoralist populations described by Dutch colonists in the 17th century—initially referred to as “Hottentots”—were Khoekhoe-speakers. They are usually referred to as the Cape-Khoekhoe and !Ora people [who were previously indicated as Korana (Barnard 1992)], but their genetic relationships with other extant populations are obscure, as they became “extinct” soon after the arrival of the Europeans. Little is also known about the Taa speaking populations that inhabited the Southernmost area of southern Africa, such as the /Xam, the /Xegwi, and the Baroa (the latter sometimes referred to as the mountain bushmen, located in and around the Maloti/Drakensberg mountain range in South Africa/Lesotho), who probably spoke a language similar to the Khomani (of the !Ui group), and who were soon assimilated into Bantu populations who settled in the area. The Karretjie people of South Africa are often considered as the descendants of the /Xam. Given this complex process of contacts and admixture, it is expected that the analysis of admixed populations may help to revive the genetic ancestry of such “vanished” communities, and therefore to provide a description of the genomic landscape predating the arrival of Bantu speaking populations and European colonists.

Our analysis provides insights into the unsolved histories described above, and more generally on the populations living in the region. First, all of the approaches exploited here point to the existence of an ancient tripartite genetic structure in southern Africa populations, dating back to around 25 KYA (18–32 KYA, Figure 1B, Figure 2, Figure 3, Figure S3, and Figure S5); these dates are in line with previous estimates for the separation of the two Khoesan components (Pickrell *et al.* 2012; Schlebusch *et al.* 2012; Kim *et al.* 2014), and close to the start of Marine Isotope Stage 2, and the beginning of the Last Glacial Maximum, whose impact on the distribution of resources might have triggered such differentiation (Mitchell 2002). *Northern* Khoesan mainly comprises Ju|’Hoan and !Xun individuals, who live in the Northern Kalahari area. TREEMIX and PCA suggest that these two populations are modestly distinct from each other, underlining further structure within this component (Figure 2, B and C). Interestingly, the Khoe-Kwadi-speaking Khwe, whose genetic ancestry is mostly Bantu-related, and the Hai||om, share Khoesan genetic affinity with these populations, as expected given their geographic proximity (Figure 3A). Their genomes also contain the *Central* Khoesan component, which suggests that further admixture with populations with such ancestry may have occurred.

The *Central* Khoesan component, common in groups from the Central Kalahari, includes all the Taa populations, except the Khomani (*Southern* Khoesan), as well as the West and East Khoe-Kwadi speakers and the K’xa speaking population = Hoan. This further highlights the mismatch between genetics and linguistic affiliation in populations from the region (Schlebusch *et al.* 2012). This component has not been reported before, although Schlebusch *et al.* (2012) mentioned the unique behavior of G|ui and G||ana individuals. The inclusion of a more representative set of populations in the current analysis, a few of which are characterized by this Khoesan component, together with a focus on the Khoesan-specific genetic components has led to the secure identification and further characterization of this key element of Khoesan-related ancestry.

The *Southern* Khoesan component is represented mainly by a set of linguistically heterogenous, but geographically proximate, populations: the Khomani (Taa speakers), Karretjie, and Nama (Khoe-Kwadi). All of these populations are thought to have originated in the Northern Cape (Barnard 1992). Barnard considered “the Khoekhoe and the Bushmen [of the Cape area] as members of a single regional unit, separate from the other (black and white) peoples of the subcontinent” (Barnard 1992). This is in agreement with our findings of substantial genetic similarities between these groups, despite their different cultural affiliations. In addition, we found evidence for admixture with Eastern Africa or Eurasian sources ∼1160−1740 years ago in all the Khoesan populations, suggesting that the arrival of pastoralism happened at the same time across the whole subcontinent (Breton *et al.* 2014; Pickrell *et al.* 2014).

Taken together, our results suggest that cultural diffusion—in the absence of significant gene-flow—might have played an important role in the spread of pastoralism and possibly Khoe languages in southern Africa (Sadr 1998; Barnard 2007; Barham and Mitchell 2008; Schlebusch *et al.* 2012). The Khoesan-like genetic ancestry of the Khoe-Kwadi speaking Damara maps to the *Southern* component, which is consistent with their long-term interaction with the Nama, who speak a very similar language (Guldemann and Fehn 2014), and possibly coupled with gene-flow from K’xa populations living in the same area (as suggested by the occurrence of the *Northern* Khoesan component in their genetic make-up). All the Coloured and the Bantu populations from the Southernmost part of the continent (South Africa and Lesotho) are characterized by the *Southern* Khoesan component (Figure 3A), suggesting an overall broad homogeneity in Khoesan ancestry over this specific region. However, it is worth noting that several Bantu individuals in the LAMDS plot are slightly deviated toward central Khoesan populations, and that Bantu populations show substantial differences when compared to Coloured individuals, as our cluster analyses based on MDS and IBS distances suggest. Moreover, consistent differences in admixture times and sources have been detected among the two groups. Given their different geographical distribution, such observations could be explained by the existence of additional Khoesan structure in the region and the past presence of differentiated groups around the Lesotho/Drakensberg area (assimilated by local Bantu speaking groups), or by admixture between Bantu and *Central* or *Southern* Khoesan (Busby *et al.* 2016).

Interestingly, the tripartition observed in the Khoesan ancestry does not recapitulate cultural affiliation (Figure 3C). As described above, we in fact identified a broad inconsistency between genetic clustering and linguistic or subsistence affiliation (Pickrell *et al.* 2012). When we predicted genetic similarity among individuals from geography, predictive error was substantially lower than that of subsistence strategy or linguistic affiliation, both marginally improving the predictive power when considered together with geography. Extensive admixture and cultural transition appears to have characterized populations from this area. Similar scenarios have been proposed also for Europe (Lazaridis *et al.* 2014; Haak *et al.* 2015) and Madagascar (Pierron *et al.* 2014), suggesting a common process across human populations. The importance of geography on the distribution of genetic variation among Khoesan is further confirmed by the geogenetic map inferred by SpaceMix inferred using random prior coordinates, which recapitulates the geographic location the populations.

Our ADMIXTURE analysis of Niger-Congo-speaking populations (which includes Bantu speakers) identified four different ancestral components broadly consistent with their geographic location (Figure 1B). Specifically, we identified three Bantu components that are present in Eastern, South-Eastern and Western Africa. Interestingly, the latter is modal in the Damara, and in the pastoralist Bantu-speaking Herero and Himba (from 55% in the Himba to 86% in the DamaraP sample), but not in other Bantu-speaking groups of the region (Mbukushu ∼20%, Owambo ∼27%, and Kwangali ∼22%). This component is slightly more related to West Africa than the Eastern and South-Eastern Niger-Congo components, and its differential distribution among Bantu groups in this region may relate to different waves of Bantu colonists into southern Africa, as suggested in a recent survey of African genetic history based on haplotype analyses (Diamond 1997; Busby *et al.* 2016). Alternatively, this could simply reflect the specific and shared demographic history of the Herero, the related Himba, and the admixed Damara.

### Conclusions

The genetic structure of southern African populations is complicated by the existence of ancient population structure, onto which several layers of additional genetic ancestries have been overimposed over the last few centuries. Here, we demonstrate that local ancestry approaches can be used to tease apart the genetic structure of such ancient components, characterizing their relationships and current distribution, further supporting a role for widespread admixture in human history (Patterson *et al.* 2012; Hellenthal *et al.* 2014; Busby *et al.* 2015; Montinaro *et al.* 2015). Further insights are expected to be collected by the molecular investigation of archaeological human remains (Morris *et al.* 2014; Llorente *et al.* 2015). Beyond the obvious historical and archaeological implications for the reconstruction of the subcontinent dynamics, these observations are of relevance for anthropological studies as well as for epidemiological and translational applications (for example, in the design of genome-wide association studies).

## 
